# No cupid, just an arrow: a penetrating injury into the interventricular septum

**DOI:** 10.1186/s13019-024-02512-5

**Published:** 2024-02-03

**Authors:** Miia Lehtinen, Antti Nykänen, Peter Raivio

**Affiliations:** 1 Department of Cardiac Surgery, Heart and Lung Center, Helsinki University Hospital, Haartmaninkatu 4, Helsinki, 00290 Finland; 2 Faculty of Medicine, University of Helsinki, Helsinki, Finland

**Keywords:** Cardiac injury, Foreign body, Occupational injury

## Abstract

**Background:**

Penetrating cardiac injuries are rare but often fatal, with 16–55% mortality. We report a patient who suffered a non-fatal occupational cardiac injury.

**Case presentation:**

A 47-year-old man was operating an ironworker machine. A thin 3-cm metal fragment catapulted from the machine piercing the chest wall and the right ventricular outflow tract (RVOT), burrowing into the interventricular septum (IVS). The patient remained hemodynamically stable and walked to the nearest hospital. ECG-gated computed tomography revealed the exact location of the fragment within the IVS, allowing for detailed preoperative planning. The fragment was removed through a sternotomy and an incision through the RVOT. The postoperative course was uneventful.

**Conclusions:**

This case underscores the value of detailed preoperative imaging and the wide spectrum of clinical scenarios of penetrating cardiac injuries.

## Background

Of all penetrating thoracic injuries, only 6–12% involve the heart [1, 2] and are typically linked with gunshots and stabbing [2, 3]. Hospital mortality remains 16–55% [1–3]. Unfortunately, less than 4% of patients ever make it to the hospital [4]. We report a case of a penetrating cardiac injury in a stable patient with minimal symptoms.

## Case presentation

In a metal factory, a 47-year-old male was operating a hydraulic ironworker machine to punch holes into thin steel plates (25 × 10 × 6 mm). He wore protective goggles and a shield to guard against metal fragments. Suddenly, a fragment escaped the protective shield. Simultaneously, a powerful shock wave and pain hit the patient’s chest. The pain subsided quickly, so he continued working. After some time, he began to feel nauseous and dizzy but recovered swiftly. Concerned, he walked to the local primary care facility one kilometer away.

**Fig. 1:**
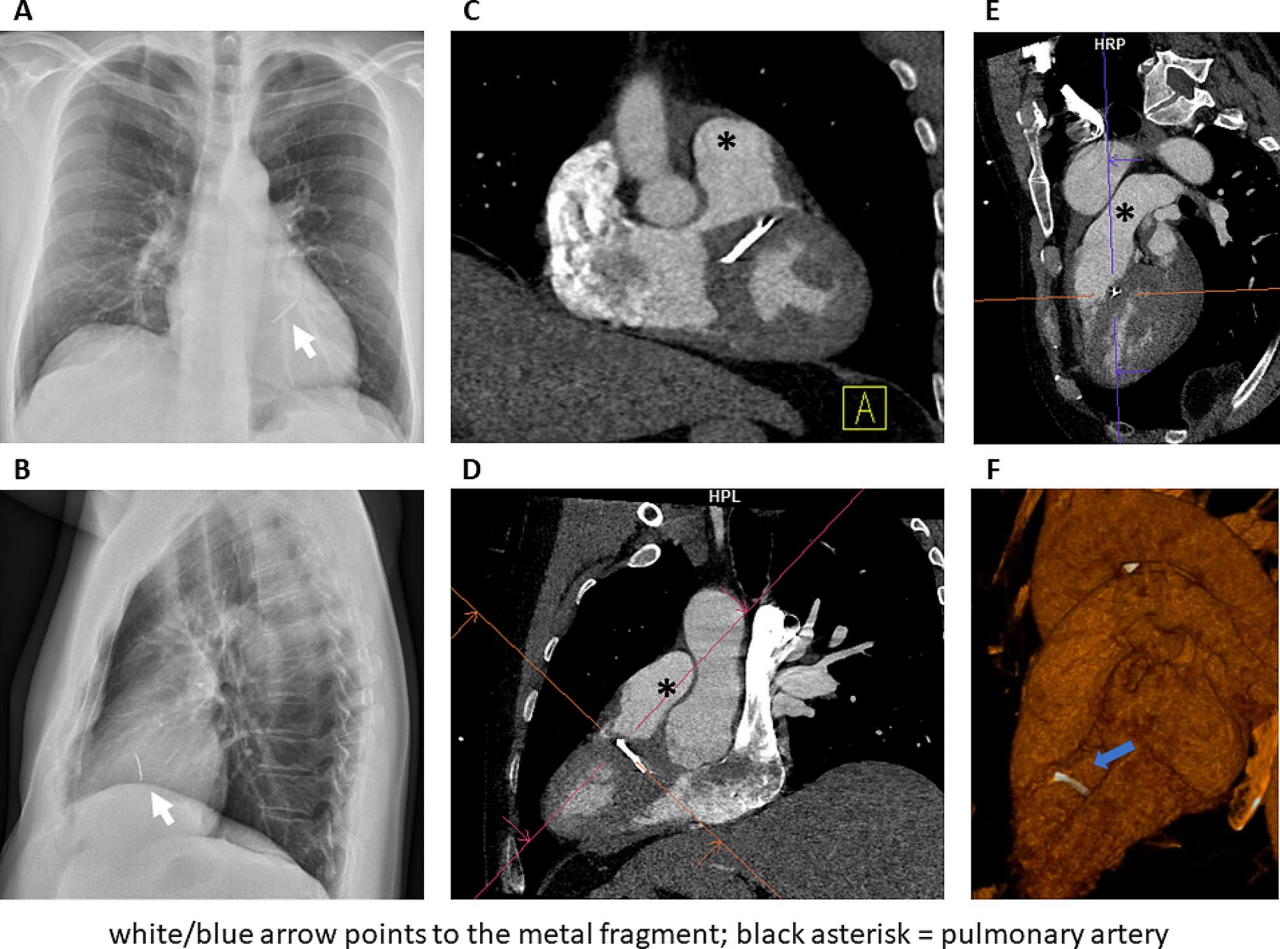
Chest X-ray (**A-B**) and ECG-gated CT scan (**C-F**) demonstrating the metal fragment in the interventricular septum

Upon arrival, his blood pressure was 117/88 mmHg, pulse rate 66/min, and oxygen saturation 98%. A tiny 3-mm wound was noticed approximately midway between his left clavicle and left areola, within the cardiac box. The patient was breathing and moving without any discomfort. A chest X-ray revealed a slim foreign body projecting over the cardiac silhouette (Fig. 1). A non-gated computed tomography (CT) scan confirmed an approximately 30 mm long piece of metal within the heart.

The patient was transferred to Helsinki University Hospital, a quaternary center with cardiac surgery service. During the transfer, he remained completely stable, in sinus rhythm, and required no analgesia or any other medication.

A transthoracic echocardiography revealed a 5-mm layer of pericardial fluid. An electrocardiogram (ECG) -gated CT scan (Fig. 1) defined more details. The fragment was 30 mm long, 4 mm thick and it was located within the infundibular interventricular septum (IVS), with its cranial tip close to the intraluminal surface of the septum in the right ventricular outflow tract (RVOT). The fragment was completely confined within the myocardium. Based on these landmarks provided by CT, we decided that the simplest and thus safest approach was to remove the fragment through a short RVOT incision.

**Fig. 2:**
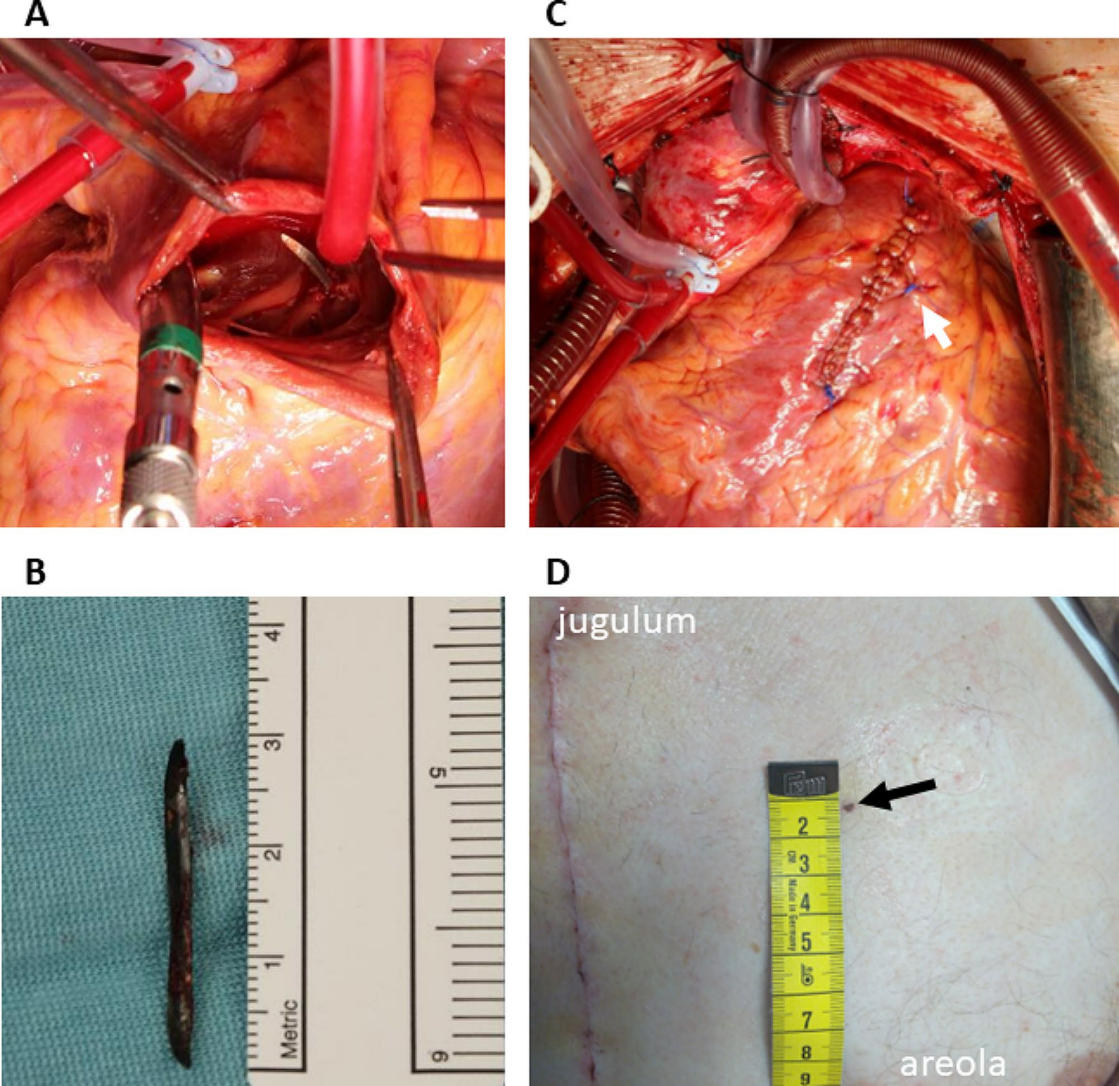
Perioperative findings. (**A**) The metal fragment revealed through a right ventriculotomy and an incision onto the interventricular septum. (**B**) The removed fragment. (**C**) The sutured entry wound in the RVOT wall (arrow) visible next to the ventriculotomy suture line. (**D**) The entry wound on the chest wall (arrow)

After informed consent, removal of the metal fragment through a sternotomy was agreed on. Perioperative transesophageal echocardiography confirmed an increased amount of pericardial fluid (a 10-mm layer), normal ventricular function and no sign of intercavitary shunts. The location of the fragment was less clearly visible in the TEE images; thus, preoperative CT guided the surgical approach. Pericardiotomy yielded 300 ml of bloody fluid; no active bleeding was detected. After heparinization and aorto-bicaval cannulation, cardio-pulmonary bypass was initiated, the ascending aorta clamped, and antegrade cardioplegia administered. Small entry wounds in the pericardial sac and the epicardium of the RVOT were detected. The RVOT was opened through a 4-cm ventriculotomy. No metal fragments were visible in the lumen. A small entry tear was observed in the muscle of the IVS as expected based on preoperative imaging. A 1 cm long superficial incision was made in the IVS to expand the entry tear and to expose the tip of the metal fragment in the septal muscle (Fig. 2). The fragment was easily slid out. The septal defect, the RVOT incision, and the epicardial entry wound were closed with sutures. Drains were left in the left pleural cavity, pericardium, and mediastinum. The patient was weaned off bypass, the sternotomy closed, and the patient was transferred to the intensive care unit.

Postoperatively, a partial right bundle branch block was observed. No arrhythmias were detected. On echocardiography on postoperative day 5, the IVS was intact, the myocardial function normal, and no pathological pericardial effusion visible. The patient was discharged on postoperative day 6.

## Discussion and conclusions

Foreign bodies retained within heart structures have been reported sporadically. Two recent publications summarized reports from numerous centers [5, 6]. Both conclude that a foreign body causing complications (hemodynamic instability, tamponade, arrhythmia) demands surgical removal, but treatment of asymptomatic patients remains controversial. Delayed complications, such as late tamponade or foreign body embolism, may arise with the heart’s movement, dislocating the object from its original location. On the other hand, surgical removal of a small < 10 mm object well embedded within the cardiac muscle may cause more tissue trauma than the object itself [7]. Multiple patients have been monitored without intervention for even decades [5].

The metal fragment in our patient was located within continuously contracting muscle. To prevent migration of the fragment and possible further injury, surgical removal was decided upon. Given the proximity of the fragment to the anterosuperior epicardial surface, it was deemed relatively simple to remove. An ECG-gated CT allowed for detailed preoperative planning.

All cardiac injuries are potentially fatal. Small foreign bodies penetrating the heart represent a subgroup with a wider spectrum of symptoms, including no symptoms, and likely carry a more optimistic prognosis. The risk of developing delayed complications supports surgical removal in selected asymptomatic patients.

## Data Availability

Not applicable.

## Abbreviations

CT: Computed tomography
ECG: Electrocardiogram
IVS: Interventricular septum
RVOT: Right ventricular outflow tract
